# Phylogenetic divergence drives distinct antimicrobial susceptibility profiles and resistomes in *Stenotrophomonas*

**DOI:** 10.3389/fmicb.2026.1805193

**Published:** 2026-03-30

**Authors:** Boqing Xu, Ruibai Wang

**Affiliations:** National Key Laboratory of Intelligent Tracking and Forecasting for Infectious Diseases, National Institute for Communicable Disease Control and Prevention, Chinese Center for Disease Control and Prevention, Beijing, China

**Keywords:** antibiotic resistance, determinant, genetic lineage, SmeDEF, *Stenotrophomonas*

## Abstract

**Background:**

*Stenotrophomonas* is a genus of increasing clinical and industrial importance, yet comprehensive antimicrobial susceptibility data and the major influencing factors are lacking, especially for species beyond *Stenotrophomonas maltophilia*.

**Methods:**

In this study, the antibiotic susceptibility of 21 average nucleotide identity (ANI) species that were relatively evenly distributed across the genus-level ANI clustering tree was tested, and the relationship between the antibiotic resistance gene profiles and phylogenetic divergence was investigated.

**Results:**

Among the 17 drugs tested, tigecycline showed 100% susceptibility, followed by chloramphenicol and trimethoprim-sulfamethoxazole (7.69% resistance). Cefotaxime had the highest resistance (97.44%), while ceftazidime and ceftazidime/avibactam showed 41.03% resistance. The overall multidrug resistance (MDR) rate was 74.36%. All strains harbored the SmeTDEF and AdeFGH multidrug efflux systems, while Smc-sgn strains commonly carried class A and B β-lactamases (*blaL1* and *blaL2*), aminoglycoside resistance genes *aph(3’)-IIc_1*, *aph(6)*-Smalt, *aph(9)-Ic*), and quaternary ammonium compound resistance genes (*qacG/J*). MDR phenotypes, resistance gene distributions, and integrity of the *smeTDEF* operon differed significantly between Smc-sgn and non-Smc-sgn strains, aligning closely with the clades resolved in the ANI-based phylogenetic tree.

**Conclusion:**

Based on this study, preliminary insights into the comprehensive antibiotic resistance profiles and genetic mechanisms of *Stenotrophomonas* have been gained. Unlike *S. maltophilia*, MDR and resistance to β-lactam antibiotics are not universal characteristics across the genus. Genetic clade background may serve as the primary determinant and driving force of multidrug resistance within this genus. Tigecycline, chloramphenicol, and SXT can be considered as broad-spectrum options for emergency or empirical treatment of *Stenotrophomonas* infections and should be prioritized in future clinical data collection to inform antibiotic treatment guidelines.

## 1 Introduction

*Stenotrophomonas* is a Gram-negative bacterium genus of the family *Lysobacteraceae* widely present in natural environments. *Stenotrophomonas* promotes plant growth through the production of auxins and hydrogen cyanide, as well as the solubilization of phosphate and potassium salts, and nitrogen fixation (Kumar et al., 2023). *Stenotrophomonas* can induce the accumulation of plant hormones like jasmonic acid and produce active bactericidal factors, including proteases, serine proteases, and R-type bacteriophage tail-like bacteriocins to combat crop pests such as the beet armyworm (*Spodoptera exigua*) and biocontrol of plant diseases such as potato *Fusarium* wilt and gray mold (Liu et al., 2013). Furthermore, *Stenotrophomonas* aids in the bioremediation of soil by enzymatically degrading various industrial and agricultural pollutants or wastes, including chemical pesticides and feathers. As a result, it holds significant emerging value for applications in both industry and agriculture.

*Stenotrophomonas maltophilia*, the first and most representative species of the genus *Stenotrophomonas*, is a common opportunistic pathogen in clinical settings. According to 2024 surveillance data from the China Antimicrobial Surveillance Network (CHINET^1^), *S. maltophilia* ranks ninth among all clinically isolated bacteria across all sample types, with an isolation rate of 2.8%, and eighth specifically in respiratory tract samples, with an isolation rate of 5.2%. This pathogen can cause infections in various tissues and organs with an attributable mortality rate ranging from 24% to 58%. Notably, the mortality rate associated with *S. maltophilia* bacteremia reaches as high as 65% (Mojica et al., 2022). As a result, *S. maltophilia* infection has been recognized as an indicator of pulmonary function deterioration and life-threatening disease in critically ill populations, including intensive care unit (ICU) patients, cancer patients, and immunocompromised individuals.

Currently, multidrug-resistance (MDR) stands out as the most prominent characteristic in the understanding of *S. maltophilia*. The World Health Organization (WHO) has classified it among the globally most concerning emerging MDR bacteria (Brooke, 2021) and the Infectious Diseases Society of America has included *S. maltophilia* in two guidelines for the hard-to-treated drug-resistant infections, alongside extended-spectrum β-lactamase–producing *Enterobacterales*, carbapenem-resistant *Enterobacterales*, *Pseudomonas aeruginosa*, *ampC* β-lactamase-producing *Enterobacterales* and carbapenem-resistant *Acinetobacter baumannii* (CRAB) (Tamma et al., 2022). MDR not only significantly complicates clinical treatment and management of *S. maltophilia*, but also poses a major obstacle to its safe bioutilization (Gil-Gil et al., 2020; Sanchez, 2015).

In addition to *S. maltophilia*, an increasing number of both known and novel species within the genus *Stenotrophomonas* have been isolated from clinical and natural environments. Examples include *S. rhizophila*, *S. sepilia*, *S. riyadhensis*, *S. pigmentata*, and *S. tuberculopleuritidis* (Yue et al., 2023). Phylogenomic analysis using genomes from the NCBI database indicates that, beyond the 39 validly named species, the genus *Stenotrophomonas* comprises a total of 116 species delineated by 95% average nucleotide identity (ANI) (Yu and Wang, 2024). However, research on antimicrobial resistance profiles and underlying mechanisms has predominantly focused on *S. maltophilia* (Gil-Gil et al., 2020; Sanchez, 2015; Brooke, 2021), with limited data available on other species and the genus. Moreover, even for *S. maltophilia*, therapeutic options and clinical data remain relatively scarce and somewhat conflicting (Tamma et al., 2022). In this study, strains from 21 ANI species—including nine validly named species—were analyzed to obtain more comprehensive insights into the antibiotic resistance profiles and genetic mechanisms of *Stenotrophomonas*.

## 2 Materials and methods

### 2.1 Strains

For *S. maltophilia*, the well-documented species of the genus *Stenotrophomonas*, three strains were selected in this study, including the reference strain 11066 (Yue et al., 2023). Our preliminary study showed that isolates belonging to the same species, collected from the same sampling site over consecutive years, maintained stable and consistent resistance profiles. For such redundant isolates, only one representative strain or type strain was included in this study. Accordingly, a total of 39 *Stenotrophomonas* strains, assigned to 21 ANI-defined species and relatively evenly distributed on the ANI-based clustering tree of the genus (Yu and Wang, 2024), were analyzed in the present study (Table 1). All strains have been subjected to next-generation genome sequencing, and species identification was performed by calculating ANI against systemic reference genomes (Yu and Wang, 2024). All strains are preserved in the strain library of the tuberculosis department of the National Institute for Communicable Disease Control and Prevention.

**TABLE 1:** Strains information and the antibiotic susceptibility testing (AST) results.

Species name	ANI species	Strain	Isolation source	MIC (μ g/mL)							
				AMP	A/S2	MERO	ETP	TAZ*	CZA	FOT	AZI
—	1	C18	E	32^R^	8/4	<0.12	<0.25	<0.25	<0.25/4	16	<2
*S. pigmentata*	9	610A2^T^	H	>32^R^	>32/16^R^	>2^R^	8^R^	>16^R^	>8/4^R^	>16^R^	8
—	22	C12	E	8	8/4	0.25	<0.25	1	1/4	16^R^	8
		Z10HUANG	E	16	16/8	1	0.5	1	0.5/4	16^R^	8
		Z18H	E	8	4/2	0.25	<0.25	1	2/4	16^R^	8
*S. nitritireducens*	24	C13	E	>32^R^	>32/16^R^	>2^R^	>8^R^	4	2/4	>16^R^	32^R^
		Z6	E	<2	<2/1	<0.12	<0.25	<0.25	<0.25/4	0.5	<2
—	117	Z2	E	>32^R^	>32/16^R^	>2^R^	4^R^	0.5	0.5/4	>16^R^	4
*S. nematodicola*	31	C20	E	>32^R^	>32/16^R^	2	4^R^	4	2/4	>16^R^	8
—	34	Z10BAI	E	>32^R^	>32/16^R^	>2^R^	>8^R^	>16^R^	4/4	>16^R^	<2
—	45	Z1	E	>32^R^	>32/16^R^	>2^R^	2^R^	1	1/4	>16^R^	8
—	56	Z4	E	>32^R^	>32/16^R^	1	2^R^	8	2/4	>16^R^	<2
—	62	C15	E	>32^R^	>32/16^R^	>2^R^	>8^R^	16	>8/4^R^	>16^R^	16
		C16	E	16	4/2	>2^R^	4^R^	1	2/4	16^R^	32^R^
		Z18B	E	>32^R^	>32/16^R^	>2^R^	>8^R^	16	8/4	>16^R^	64^R^
		Z21	E	>32^R^	>32/16^R^	>2^R^	>8^R^	8	>8/4^R^	>16^R^	>64^R^
*S. tuberculopleuritidis*	66	704A1^T^	H	>32^R^	>32/16^R^	>2^R^	>8^R^	8	8/4^R^	>16^R^	>64^R^
—	68	C17	E	>32^R^	>32/16^R^	>2^R^	4^R^	2	2/4	>16^R^	<2
*S. cyclobalanopsidis*	71	Z3	E	> 32^R^	>32/16^R^	>2^R^	>8^R^	>16^R^	8/4	8^R^	4
*S. pavanii*	83	C2	E	>32^R^	>32/16^R^	>2^R^	>8^R^	4	4/4	>16^R^	16
		C7	E	>32^R^	>32/16^R^	>2^R^	>8^R^	>16^R^	>8/4^R^	>16^R^	64^R^
		Z5	E	>32^R^	>32/16^R^	>2^R^	>8^R^	4	4/4	>16^R^	8
*S. muris*	85	Z98	H	>32^R^	>32/16^R^	>2^R^	>8^R^	4	2/4	>16^R^	64^R^
		Z101	H	>32^R^	>32/16^R^	>2^R^	>8^R^	8	2/4	>16^R^	32^R^
—	87	C14	E	>32^R^	>32/16^R^	>2^R^	>8^R^	1	1/4	>16^R^	32^R^
—	91	Z140	E	>32^R^	>32/16^R^	>2^R^	>8^R^	>16^R^	>8/4^R^	>16^R^	>64^R^
*S. maltophilia*	91	11066	H	>32^R^	>32/16^R^	>2^R^	>8^R^	>16^R^	>8/4^R^	>16^R^	64^R^
		C6	E	>32^R^	>32/16^R^	>2^R^	>8^R^	>16^R^	>8/4^R^	>16^R^	64^R^
		C8	E	>32^R^	>32/16^R^	>2^R^	>8^R^	>16^R^	>8/4^R^	>16^R^	32^R^
—	92	C4	E	>32^R^	>32/16^R^	>2^R^	>8^R^	4	4/4	>16^R^	32^R^
		C19	E	>32^R^	>32/16^R^	>2^R^	>8^R^	8	2/4	>16^R^	64^R^
*S. geniculata*	98	Z64-7	E	>32^R^	>32/16^R^	>2^R^	>8^R^	>16^R^	>8/4^R^	>16^R^	64^R^
		Z40-10	E	>32^R^	>32/16^R^	>2^R^	>8^R^	>16^R^	>8/4^R^	>16^R^	64^R^
		C45	H	>32^R^	>32/16^R^	>2^R^	>8^R^	>16^R^	>8/4^R^	>16^R^	<2
		C3	E	>32^R^	>32/16^R^	>2^R^	>8^R^	>16^R^	>8/4^R^	>16^R^	>64^R^
		C5	E	>32^R^	>32/16^R^	>2^R^	>8^R^	>16^R^	>8/4^R^	>16^R^	>64^R^
—	102	G28-11	E	>32^R^	>32/16^R^	>2^R^	>8^R^	>16^R^	>8/4^R^	>16^R^	64^R^
—	104	C9	E	>32^R^	>32/16^R^	>2^R^	>8^R^	>16^R^	>8/4^R^	>16^R^	64^R^
		C11	E	>32^R^	>32/16^R^	>2^R^	>8^R^	>16^R^	>8/4^R^	>16^R^	64^R^
**MIC (μ g/mL)**										**Number of resistant**	
CIP*		**NAL**	**AMI**	**STR**	**TET**	**TGC**	CHL*	**COL**	SXT*	**Drug classes**	
0.5		8	8	>32^R^	8	<0.25	<4	<0.25	<0.5/9.5	2	
>2^R^		>32^R^	<4	16	8	0.5	16	<0.25	>8/152^R^	3	
0.25		<4	16	>32^R^	<1	<0.25	<4	>8^R^	1/19	3	
0.25		<4	8	32^R^	<1	<0.25	<4	>8^R^	1/19	3	
0.25		<4	8	32^R^	<1	<0.25	8	>8^R^	4/76	3	
2		8	>64^R^	>32^R^	16^R^	0.5	16	>8^R^	<0.5/9.5	5	
0.12		8	<4	16	16^R^	0.5	<4	0.5	>8/152^R^	2	
0.5		<4	8	>32^R^	2	<0.25	16	<0.25	<0.5/9.5	2	
1		<4	8	>32^R^	<1	<0.25	<4	>8^R^	<0.5/9.5	3	
0.5		<4	<4	16	<1	<0.25	<4	<0.25	<0.5/9.5	1	
0.5		<4	8	>32^R^	2	<0.25	8	<0.25	<0.5/9.5	2	
1		<4	8	32^R^	2	<0.25	<4	<0.25	1/19	2	
2		16	>64^R^	>32^R^	16^R^	1	16	<0.25	<0.5/9.5	3	
2		<4	>64^R^	>32^R^	16^R^	0.5	16	>8^R^	2/38	5	
2		16	>64^R^	>32^R^	16^R^	0.5	16	>8^R^	1/19	5	
2		<4	>64^R^	>32^R^	8	<0.25	16	>8^R^	<0.5/9.5	4	
>2^R^		8	64^R^	>32^R^	16^R^	0.5	16	8^R^	8/152^R^	7	
1		<4	<4	<8	4	<0.25	8	<0.25	<0.5/9.5	1	
2		32^R^	<4	16	4	<0.25	16	<0.25	<0.5/9.5	2	
2		8	>64^R^	>32^R^	16^R^	0.5	16	>8^R^	<0.5/9.5	4	
1		<4	>64^R^	>32^R^	>16^R^	0.5	16	>8^R^	<0.5/9.5	5	
1		<4	32	>32^R^	8	<0.25	<4	0.5	<0.5/9.5	2	
2		8	>64^R^	>32^R^	16^R^	0.5	16	>8^R^	<0.5/9.5	5	
2		8	>64^R^	>32^R^	16^R^	0.5	16	>8^R^	<0.5/9.5	5	
0.5		<4	64^R^	>32^R^	4	<0.25	8	<0.25	<0.5/9.5	3	
>2^R^		16	>64^R^	>32^R^	16^R^	1	16	>8^R^	<0.5/9.5	5	
>2^R^		8	>64^R^	>32^R^	>16^R^	0.5	8	>8^R^	<0.5/9.5	5	
2		<4	>64^R^	>32^R^	>16^R^	0.5	8	>8^R^	< 0.5/9.5	5	
2		<4	32	>32^R^	16^R^	0.5	8	8^R^	<0.5/9.5	5	
2		8	32	>32^R^	8	<0.25	<4	>8^R^	<0.5/9.5	4	
>2^R^		16	>64^R^	>32^R^	16^R^	0.5	8	>8^R^	<0.5/9.5	5	
>2^R^		16	64^R^	>32^R^	16^R^	2	32^R^	2	1/19	5	
>2^R^		16	>64^R^	>32^R^	16	1	16	>8^R^	<0.5/9.5	5	
0.12		<4	<4	16	<1	<0.25	<4	>8^R^	<0.5/9.5	2	
>2^R^		32^R^	64^R^	>32^R^	>16^R^	0.5	16	>8^R^	<0.5/9.5	6	
2		32^R^	>64^R^	>32^R^	>16^R^	1	>32^R^	>8^R^	<0.5/9.5	7	
>2^R^		32^R^	32	>32^R^	16^R^	0.5	32^R^	8^R^	<0.5/9.5	7	
>2^R^		16	>64^R^	>32^R^	>16^R^	0.5	16	>8^R^	<0.5/9.5	5	
2		<4	64^R^	>32^R^	16^R^	<0.25	8	>8^R^	<0.5/9.5	5	

*Judged by CLSI-recommended breakpoints. Drugs determined to be resistant based on antimicrobial breakpoints are marked with a superscript “R”. Antibiotics on the plates: AMP, ampicillin; A/S2, ampicillin/sulbactam; MERO, meropenem; ETP, ertapenem; TAZ, ceftazidime; CZA, ceftazidime/avibactam; FOT, cefotaxime; AZI, azithromycin; CIP, ciprofloxacin; NAL, nalidixic Acid; AMI, amikacin; STR, streptomycin; TET, tetracycline; TGC, tigecycline; CHL, chloramphenicol; COL, colistin; SXT, trimethoprim-sulfamethoxazole. Strain sources: H (patient isolates) and E (environmental isolates). —, without a validated species name.

### 2.2 Antibiotic susceptibility testing (AST)

Antibiotic susceptibility of the strains to 17 drugs commonly used for Gram-negative strains was tested using the customized AST panel for the Chinese Pathogen Identification Network (CHNENF, Trek Diagnostic Systems Ltd., West Sussex, United Kingdom). Judgment was made according to the interpretive minimum inhibitory concentration (MIC) breakpoints for *Stenotrophomonas* recommended by the Clinical and Laboratory Standards Institute [CLSI] (2023) and the instruction of the CHNENF plate (Supplementary Table 1). In this study, strains with an MIC exceeding the test concentration (>2 μg/mL) were classified as resistant. The 17 drugs belong to eight classes, and strains resistant to three or more drug classes are defined as MDR.

### 2.3 Genetic analyses of antimicrobial resistance genes

Resistance gene screening was performed on the genomes of all tested strains using Abricate (v1.0.1) with four databases, CARD, ResFinder, ARG-ANNOT (updated to 2025-Dec-01) and NCBI (updated 2025-Jan-14). Initial screening parameters were set to –minid 60 and –mincov 50 (sequence identity ≥ 60% and gene coverage ≥ 50%), and a stricter screening step was applied using awk with required identity ≥ 80% and gene coverage ≥ 90%. Specific β-lactamase genes were identified using the regular expression *blaL1*| *blaL2* and coverage ≥ 90%, and no minimum identity threshold was set. The strict screening result files for each database were then summarized.

Screening results were merged into a unified table. Deduplication based on the “genome-gene” pair was performed using associative arrays in awk to ensure that only the record with the highest sum of coverage and identity was retained for each unique gene in each genome. This deduplicated result file was subsequently used to construct a binary gene presence/absence matrix.

Visualization and analysis were conducted in R v4.1.2. A Pearson correlation matrix was calculated between genomes. The pheatmap package was used to generate a presence/absence heatmap of the genes, and hierarchical clustering based on Jaccard distance was performed to create dendrograms for both genes and samples. Principal component analysis (PCA) was applied to the binary matrix. The data were variance-scaled, and the first two principal components were extracted for visualization. The ggrepel package was employed to plot the PCA results with non-overlapping sample labels.

### 2.4 Statistical analysis

Statistical analyses, including comparisons and stratification of resistance rates, were conducted using IBM SPSS Statistics (Version 26; IBM Corp., Armonk, NY, United States).

## 3 Results

### 3.1 Antibiotic resistance profiles of *Stenotrophomonas*

**FIGURE 1:**
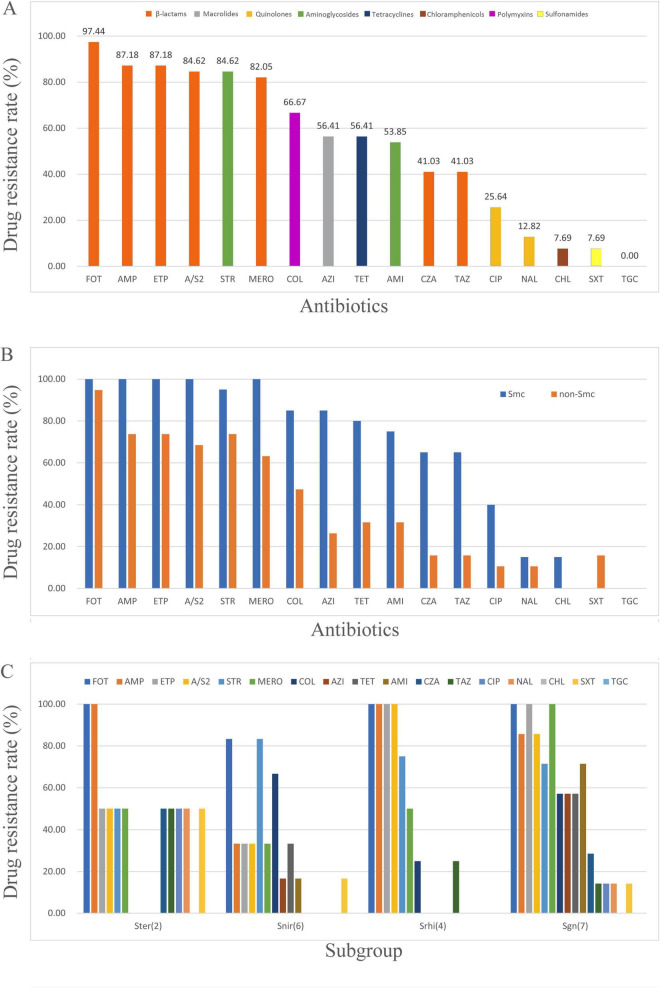
Prevalence of antibiotic resistance among the studied *Stenotrophomonas* isolates. **(A)** Overall resistance rates to 17 antimicrobial agents. **(B)** Comparison of resistance rates between the Smc and non-Smc. **(C)** Resistance rates across the four non-Smc subgroups.

Among the 17 drugs tested, all strains were sensitive to tigecycline (100%), showing the highest susceptibility. Chloramphenicol and trimethoprim-sulfamethoxazole (SXT) followed, with resistance rates as low as 7.69% (Figure 1A). The tested strains exhibited high resistance rates to β-lactam antibiotics. Among the six β-lactams tested, cefotaxime had the highest resistance rate (97.44%), while ceftazidime and ceftazidime/avibactam, which are also cephalosporins, showed the lowest resistance rates among β-lactams (41.03%) and their resistance rates were even lower than that of aminoglycosides, azithromycin and tetracycline (Figure 1A). Furthermore, the results indicated that the β-lactamase inhibitors sulbactam and avibactam were effective in some *Stenotrophomonas* strains, reducing the MICs of ampicillin and ceftazidime, respectively, although their impact on overall resistance rates remained limited (Table 1).

On the ANI-based phylogenetic tree, aside from *S. maltophilia* complex (Smc, ANI species 74–106, represented by *S. maltophilia*) defined by the 90% ANI threshold, the non-Smc species can be divided into Snir (ANI species 19–26, 117, represented by *S. nitritireducens*), Ster (ANI species 1–28, represented by *S. terrae*), Srhi (ANI species 31–59, represented by *S. rhizophila*), and Sgn (ANI species 60–73, including Sgn1, Sgn3, and Sgn4). Among these, Sgn and Smc are most closely related and located on the same major branch (Yu and Wang, 2024). AST results showed that the resistance of Smc strains is significantly more severe than that of non-Smc strains, with Sgn showing the highest level of resistance among the non-Smc strains (Figures 1B, C).

Among the 17 antibiotics tested, representing nine distinct classes, 29 strains (74.36%) were classified as multidrug-resistant (MDR), with the widest resistance spectrum covering up to seven classes. The prevalence of MDR was significantly higher in Smc strains (18/20, 90.00%) than in non-Smc strains (11/19, 57.90%) (Fisher’s exact test, *p* = 0.034). No significant difference was observed in the overall MDR rate between human-derived and environmental isolates (5/6, 83.33% vs. 24/33, 72.72%, Fisher’s exact test, *p* = 0.659). However, stratified analysis revealed notable effect modification by Smc/non-Smc subgrouping: within the Smc, environmental isolates exhibited a higher MDR rate than human-derived isolates (15/16, 93.75% vs. 3/4, 75.00%), whereas the opposite trend was seen among non-Smc isolates (9/17, 52.94% vs. 2/2, 100.00%). After adjusting for subgroup, the Cochran–Mantel–Haenszel test confirmed the absence of a significant association between strain source and MDR status (*p* = 0.847).

Notably, the only non-MDR human-derived strain, C45, showed high resistance to all β-lactams and was also resistant to colistin. While some *Stenotrophomonas* strains indeed demonstrated relatively low resistance profiles. For instance, Z10BAI and C17 were resistant only to certain β-lactam antibiotics, while Z6 (*S. nitritireducens*), despite being resistant to tetracycline and SXT, remained susceptible to all other antibiotics, including the entire β-lactam class—a high sensitivity pattern previously unreported in *Stenotrophomonas*.

### 3.2 Antibiotic resistance genes

**FIGURE 2:**
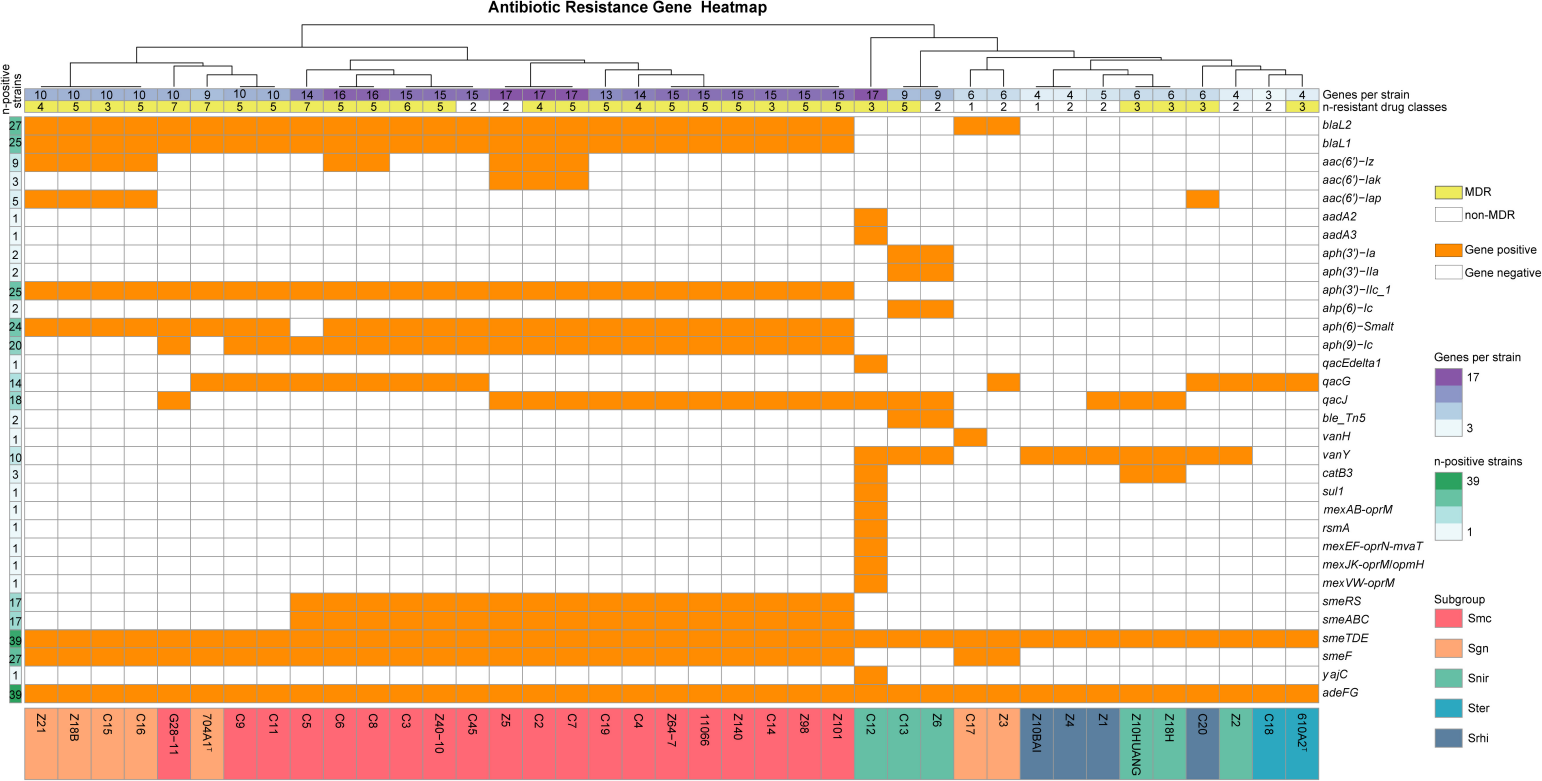
Clustered heatmap based on the similarity of their antibiotic resistance gene profiles among the studied strains.

Resistance gene screening identified that the tested strains collectively harbored 37 antimicrobial resistance genes (Figure 2). Among the 12 aminoglycoside resistance-related genes, there were three *aac(6’)* subtypes —*aac(6’)-Iz*, *aac(6’)-Iak*, and *aac(6’)-Iap* —of encoding *S. maltophilia*-derived aminoglycoside acetyltransferase (Lambert et al., 1999; Lund et al., 2023), along with two aminoglycoside-3″-O-adenylyltransferase genes (*aadA2* and *aadA3*) and seven variants belonging to three classes of aminoglycoside phosphotransferases [*aph(3’)*, *aph(6)*, and *aph(9)*]. Within the β-lactamase genes identified, those encoding class A serine β-lactamases (*blaL2*) and species-specific class B metallo-β-lactamases L1 (*blaL1*), the critical resistance marker of *S. maltophilia* were present. Additionally, other direct resistance determinants were present, such as *qacG* and *qacJ* (conferring low-level resistance to quaternary ammonium disinfectants), *ble_*Tn5 (associated with bleomycin resistance), *vanH* and *vanY* (involved in vancomycin resistance), and *catB3* (a variant of the *cat* gene conferring resistance to chloramphenicols). Among these directly drug resistance-related genes, *blaL2* and *blaL1* exhibited the highest carriage rates (69.23% and 64.10%, respectively, Figure 2). The carriage rates of the aminoglycoside resistance-related genes *aph(3’)-IIc_1* and *aph(6)-Smalt* (61.54%) were second only to those of the β-lactamase genes. The carriage rate of *catB3* was 7.69%, which corresponded exactly to the observed phenotypic resistance rate to chloramphenicol.

In *Stenotrophomonas*, powerful efflux pump systems play a more fundamental and critical role in contributing to MDR and even pan-drug-resistance than acquired specific resistance genes. The SmeTDEF system—which is directly associated with significantly reduced susceptibility or outright resistance to quinolones, tetracyclines, macrolides, chloramphenicol, TMP-SMX and novobiocin (Zhang et al., 2001)—along with AdeFG (a resistance-nodulation-division efflux pump known to confer resistance to fluoroquinolones, tetracycline, tigecycline, chloramphenicol, clindamycin, trimethoprim, and sulfamethoxazole in *A. baumannii* (Cortez-Cordova and Kumar, 2011), was detected in all tested *Stenotrophomonas* strains. Interestingly, SmeDEF exists in two distinct forms within *Stenotrophomonas*. In strains belonging to the Smc-sgn group, eight genes, including the complete *smeTDEF* operon are located between the nucleoside deaminase and *hslU* genes. smeT encodes a TetR/AcrR family transcriptional regulator of this operon, while *smeDEF* encode the periplasmic adaptor, permease and outer membrane subunit of this RND-type multidrug efflux transporter, respectively. In non-Smc-sgn strains, however, only three genes—*tetR*-*smeDE*—are present in the same genomic region, with five genes, including *smeF*, being absent. The *smeABC* operon and its two-component signal transduction system *smeSR*, which confers resistance to aminoglycosides, β-lactams, and fluoroquinolones (Li et al., 2002), were detected in 85% (17/20) of the Smc strains.

Notably, besides *smeDEF* and *adeFG*, strain C12 (ANI species 22) also carries genes associated with four distinct inner membrane multidrug efflux complexes: MexAB-OprM, *rsmA*-MexEF-OprN-MvaT, MexJK-opmH, and MexVW-OprM (Figure 2). Moreover, among all 39 strains tested, C12 was the only one found to carry a Class 1 integron, which included the conserved 3’-CS marker *qacEdelta1*, along with the genes *aadA2*, *aadA3*, and *sul1*—elements typically associated with and located within Class 1 integrons. Paradoxically, despite harboring this broad resistance gene repertoire, C12 exhibited phenotypic resistance to only three antimicrobial agents: cefotaxime, streptomycin, and colistin. It remained susceptible to SXT, an antibiotic combination typically conferred by *sul* genes (Boncompagni et al., 2025). The *sul1* gene from C12 was subsequently extracted and found to be 840 bp in length, exhibiting 100% sequence identity to the *sul* gene (WP_225624817) of *Klebsiella aerogenes* with no mutations.

Clustering analysis based on similarity in resistance gene carriage revealed a clear species-specific pattern, with differences primarily observed between strains inside and outside the Smc, similar to the phenotypic resistance pattern (Figure 2). All strains within the Smc carried more than 10 resistance genes, with common genes including class A and B β-lactamases, aminoglycoside resistance genes [*aph(3’)-IIc_1*, *aph(6)-Smalt*, *aph(9)-Ic*], quaternary ammonium compound resistance genes (*qacG/J*), and efflux pumps (*smeDEF* and *adeF*). Among species outside the Smc, the closely related Sgn subgroup displayed a resistance gene profile highly similar to that of Smc, including the same complete *smeTDEF* operon and the presence of the *blaL2* gene. The main difference was the absence of *smeABC*, which was highly prevalent among Smc species. For non-Smc species other than Sgn—except for the atypical strain C12—the number of resistance genes was below 9. Besides lacking *smeABC*, these strains also did not carry class A or B β-lactamases, which is consistent with their generally higher antibiotic susceptibility.

## 4 Discussion

The AST results for multiple *Stenotrophomonas* species in this study revealed two key findings: (1) unlike *S. maltophilia*, MDR is not a common characteristic across this genus, and (2) resistance to β-lactam antibiotics is not an intrinsic trait of *Stenotrophomonas*. Within the Smc, 10% of isolates were non-MDR, whereas outside the Smc, nearly half (42.10%) of isolates were non-MDR. The susceptibility rate to ceftazidime and ceftazidime/avibactam was 35% within the Smc, but increased significantly to 84.21% outside the Smc, with 26.31% of isolates even showing susceptibility to ampicillin.

The clinical management challenge of *S. maltophilia* infection is similar to that of CRAB. Although antibiotics such as SXT, ciprofloxacin, tigecycline, ceftazidime, gentamicin, and chloramphenicol are most commonly used globally to combat *S. maltophilia* (Klimkaite et al., 2025; Tamma et al., 2022), even for this most studied and focused species within the *Stenotrophomonas* genus, data on which drugs are effective against *S. maltophilia*, the priority order of these drugs, and whether commonly used combination therapies provide additional benefits remain incomplete. Currently, there is no clear “standard treatment” antibiotic regimen available to evaluate the effectiveness of various treatment options. Based on the *in vitro* AST of this study, tigecycline, chloramphenicol, and SXT can be regarded as the preferred agents for the emergency or empirical treatment of infections caused by the entire genus *Stenotrophomonas*, including *S. maltophilia*. Tigecycline exhibited the highest susceptibility rate and is the first-choice drug, which is also widely used in China for the treatment of critically ill patients with drug-resistant bacterial infections in the ICU. Notably, chloramphenicol is more suitable for non-Smc strains, whereas SXT is more appropriate for Smc strains. The difficulty of antibacterial treatment for non-Smc strains is lower than that for Smc strains, and quinolones as well as ceftazidime can also be used for the treatment of non-Smc strains. In future clinical treatment data collection and comparisons, greater emphasis can be placed on these drugs.

Notable disparities in antimicrobial susceptibility phenotypes and genetic profiles between strains inside and outside Smc were demonstrated in this study, which underscores that genetic lineage background (Smc versus non-Smc) is the more central determinant and driving force of multidrug resistance in the *Stenotrophomonas* genus. In contrast to the reports by Klimkaite et al. (2025), Paopradit et al. (2017) but consistent with studies by Minkwitz and Berg (2001), Youenou et al. (2015) on *S. maltophilia*, our findings do not support the an effect of human versus environmental origins on the multidrug resistance profiles of the examined strains. However, owing to the small number of human-derived strains in this study, the statistical power was limited. Thus, no definitive conclusion can yet be drawn regarding the influence of isolation source on antimicrobial resistance, and further investigation is required. Nonetheless, it is clear that environmental strains also play an important role in shaping the overall resistance patterns of the *Stenotrophomonas* genus. This is exemplified by the fact that the three strains exhibiting the highest level of phenotypic resistance (resistance to ≥6 drug classes)—G28-11 (ANI species 102), C5 (*S. geniculata*), and C3 (*S. geniculata*)—were all environmental, non-*S. maltophilia* Smc isolates. Nevertheless, phylogenetic analysis based on ANI (Yu and Wang, 2024) and the similarity in antibiotic resistance gene distribution (Figure 2) both suggest that within the genus *Stenotrophomonas*, there are two evolutionary clades, and the one exhibiting high-level multidrug resistance is not limited to Smc but also includes the sgn subgroup (ANI species 60–73).

*Stenotrophomonas* species exhibit considerable variation in growth rates even in same species in this study. Strains 610A2^T^ (*S. pigmentata*) (Li et al., 2024), Z10BAI (ANI 34), Z1 (ANI 45), Z3 (*S. cyclobalanopsidis*), Z6 (*S. nitritireducens*) and three strain of ANI species 22, —namely Z10HUANG, C12, and Z18H — demonstrated slow growth, necessitating 48–72 h for reliable interpretation of AST results, rather than the typical 18–24 h. Consequently, special attention should be given when selecting commercial AST panels, and careful adjustment of endpoint time settings is essential when using automated systems such as the BD Phoenix. Furthermore, the ciprofloxacin upper concentration limit on commonly used Gram-negative commercial AST panels, including CMV4AGNF, BD Phoenix NMIC 413, the 801 Panel and et al., is typically 2 or 4 μg/mL. This is below the ≥8 μg/mL resistance breakpoint recommended by CLSI for *S. maltophilia*. Consequently, the use of these panels may lead to an overestimation of ciprofloxacin resistance rates in *Stenotrophomonas*, as observed in this study where ciprofloxacin surprisingly exhibited a higher resistance rate compared to nalidixic acid.

Finally, the AST results showed that none of the 17 antibiotics tested were resistant across all strains. Even cefotaxime, which had the highest resistance rate, was still effective against some strains, exhibiting MIC as low as 0.5 μg/mL. Therefore, any selective media formulated by adding antibiotics may fail to detect certain *Stenotrophomonas* isolates. This applies to both steno medium agar (SMA) and its modified versions, which contain amphotericin B (2.5 μg/mL), imipenem (32 μg/mL), and vancomycin (10 μg/mL) (Goncalves-Vidigal et al., 2011). Although the susceptibility of the strains in this study to these three specific antibiotics was not directly tested, the resistance rates observed for meropenem (82.05%) and ertapenem (87.18%), using the ≥4 and ≥2 μg/mL breakpoints, suggest that SMA is unsuitable for the selective isolation of *Stenotrophomonas* species.

## 5 Conclusion

This study conducted phenotypic antimicrobial susceptibility testing and resistance gene detection on 21 *Stenotrophomonas* ANI species, encompassing multiple species beyond *S. maltophilia* and Smc. These findings preliminarily offer a clearer, more comprehensive understanding of the antibiotic resistance profile and its underlying causes within the genus *Stenotrophomonas*, which also aid the evidence-based selection of clinical therapeutic agents and the formulation of a combined antibiotic regimen in simple or complicated infections.

## Data Availability

The datasets presented in this study can be found in online repositories. The names of the repository/repositories and accession number(s) can be found in the article/Supplementary material.

## Footnotes

1
https://www.chinets.com
